# Retrograde Popliteal Access for Challenging Superficial Femoral Artery Occlusion

**DOI:** 10.1155/2021/8833025

**Published:** 2021-05-17

**Authors:** Georges Ibrahim, Sami Nabhani, Michel Feghaly

**Affiliations:** Division of Vascular Surgery, Saint Georges Hospital-University Medical Center, Ashrafieh, Beirut, Lebanon

## Abstract

Retrograde popliteal access has long been established as an alternative to the antegrade approach to occlusive lesions in the superficial femoral artery (SFA). However, early reports with high complication rates (dissection, hematomas, aneurysms, and arteriovenous shunts at the puncture site) reduced enthusiasm for this technique. In recent years, with the development of thinner sheaths and low profile angioplasty devices, retrograde popliteal access has resurfaced as a viable technique, mostly in combination with or after failure of the more classical antegrade approach. In this retrospective study, we will report the safety and efficacy of the retrograde popliteal approach in the treatment of superficial femoral artery chronic total occlusions, in 13 consecutive patients between January 2017 and January 2021. The results showed 100% successful puncture of the popliteal artery and 100% successful recanalization and stenting of the superficial femoral artery with a total of 2 complications related to the puncture site and zero periprocedural mortality. In conclusion, the retrograde popliteal approach appears to be an effective and safe alternative to the common SFA complete total occlusion (CTO) treatment approach.

## 1. Introduction

Endovascular procedures are having an increased role in the treatment of peripheral arterial disease [1]. Infrainguinal procedures are usually performed using the contralateral retrograde or the ipsilateral antegrade common femoral artery access. While the contralateral retrograde access with crossover technique proves to be an easy access, the ipsilateral antegrade common femoral artery access is a more technically demanding access but with improved control [2]. In up to 20% of complex cases, there is a failure of recanalization, mainly due to inability to reenter the distal true lumen [3–5].

Various sophisticated devices have been developed for subintimal reentry, but their high costs have prohibited their widespread use [6]. Consequently, the retrograde popliteal artery approach was established as a way to increase the success rate in SFA recanalization.

The aim of this study is to assess the safety of retrograde popliteal access for SFA lesions, as well as rate the procedural success. This was defined as successfully puncturing the popliteal artery as well as angiographic success.

This technique was first described years ago by Tønnesen et al., with results of 50 angioplasty procedures via the popliteal artery. It was described as especially suited for lesions close to the takeoff of the superficial femoral artery [7].

Since then, it has diminished in popularity owing to complications, such as dissections, vessel ruptures, arteriovenous fistulas, pseudoaneurysm, and hematomas [8, 9].

Trigaux et al. described the relationship between the popliteal artery (PA) and the popliteal vein, and the safest technique to puncture the PA. Several guidance methods for PA puncture have since then been reported, notably, by ultrasound (US) guidance and digital subtraction angiography (DSA) [10].

## 2. Methods

This is a case series of 13 patients who underwent percutaneous retrograde popliteal access (RPA) for the recanalization of SFA occlusions.

### 2.1. Procedure

All patients gave written informed consent before undergoing the procedure. All procedures were done in a dedicated angiosuite under sterile conditions.

At first, all patients had a trial of recanalization using the classical approach through the common femoral artery, using either the antegrade ipsilateral approach or the retrograde contralateral approach, and RPA was only used after failure of crossing the lesion.

Recanalization using the RPA was done either at a separate scheduled procedure where the patient was put in a prone position to facilitate the popliteal puncture, or in the same setting and the patient was kept in the same supine position but with the knee slightly flexed and externally rotated. In our series, 6 patients (46%) had their procedure done in the prone position in a separate procedure than the 1^st^ attempted recanalization, while 7 (54%) were done in the supine position where femoral access and retrograde popliteal access were done.

Popliteal puncture was done under US guidance. The popliteal fossa was examined, and the popliteal artery and veins were identified using the color Doppler and compression maneuver.

Local anesthesia was injected, followed by puncturing the popliteal artery in an area where the popliteal vein was not superimposing. Introducers used ranged in size between 4 F and 6 F depending on whether recanalization, angioplasty, and stenting were all done from the popliteal access or just the concomitant femoral access was used for stent deployment.

All patients received 5000 IU of intravenous heparin.

Recanalization was done using a hydrophilic guide wire and either a 4 F or a 5 F support catheter. Intraluminal recanalization was tried at first, but subintimal recanalization was used when inevitable.

Prestenting balloon dilatation was done in all cases followed by deployment of nitinol self-expandable stents covering the entirety of the diseased vessel with at least 5 mm of disease-free vessel coverage at the proximal and distal vessels. When more than 1 stent was used, stents were overlapped by 10 mm. Poststenting balloon dilatation was done for full stent expansion.

Completion angiography was done in all cases to control for adequate flow through the recanalized SFA and popliteal artery and to rule out any distal embolization.

Postangioplasty, the popliteal access was managed with either a minimum of 15 minutes of manual compression followed by compressive dressing for 12 hours or the deployment of an extravascular closure device followed by 10 minutes of compression and a compressive dressing for 12 hours.

Periprocedural dual antiplatelet therapy was initiated with aspirin and clopidogrel for at least 1 month, followed by aspirin alone indefinitely.

### 2.2. Definitions

Technically successful PA puncture was considered when the popliteal artery was punctured with retrograde passage of the guide wire. Technical success was defined as successful guidewire passage through the SFA occlusion (intraluminal or subintimal) from the retrograde PA approach and <30% residual stenosis after the intervention.

Complications, should they occur, were death, pseudoaneurysm, hematoma, AV shunt, neuropathy, and acute thrombosis.

## 3. Results

### 3.1. Patients

From January 2017 till January 2021, in a single center, 13 cases had chronic SFA occlusions that underwent angioplasty through a RPA.

Of the 13 cases, 8 (61.5%) were males with an average age of 77.6 ± 7.5 years; 10 (77%) of the cases presented with critical limb ischemia with 9 (69%) of the patients already presenting with tissue loss; the remaining 3 patients had severe claudication limiting their daily lifestyle. The majority of the patients presented with extensive comorbidities that can be seen in Table 1. 100% (*n* = 13) had concomitant coronary arterial disease, 77% (*n* = 10) were smokers, 92% (*n* = 12) had diabetes mellitus type 2, 100% (*n* = 13) had dyslipidemia and hypertension, and 69% (*n* = 9) had chronic kidney disease.

All patients had baseline physical examination followed by ankle-brachial index (ABI) measurements (Table 2**)** and duplex ultrasound, as well as a diagnostic angiography to outline the anatomy of the vessels and define the characteristics of the lesions, seen in Table 3.

**Figure 1:**
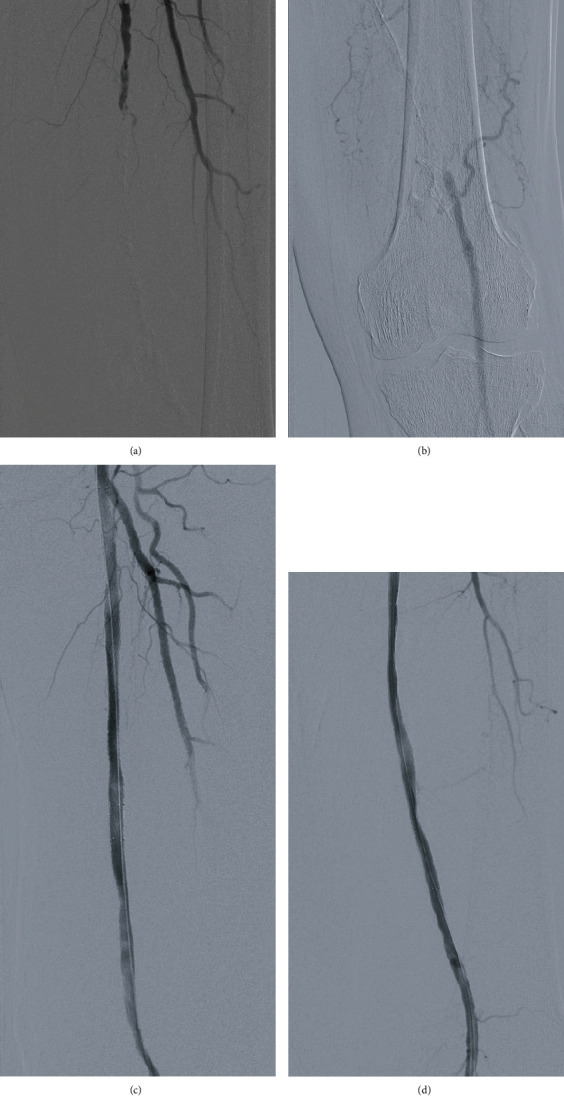
(a, b) Prerecanalization; (c, d) postrecanalization.

**Figure 2:**
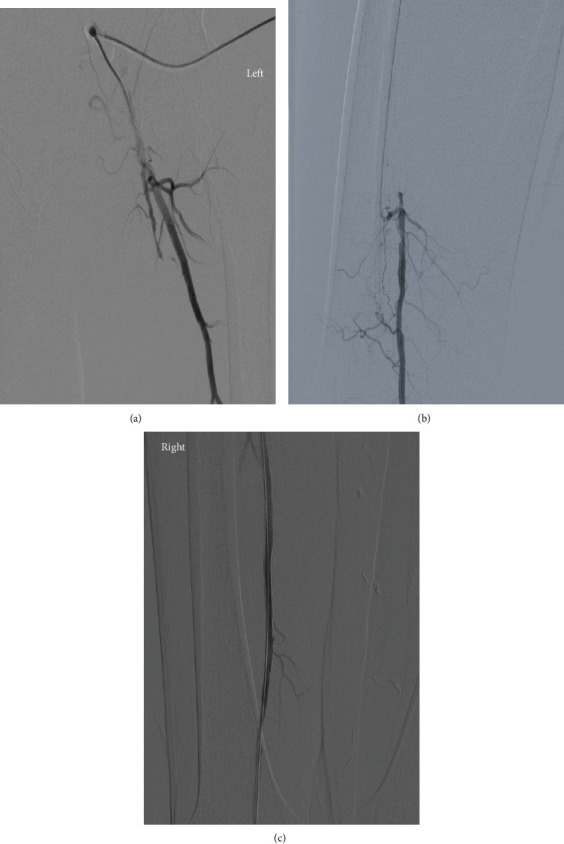
(a, b) Prerecanalization; (c) postrecanalization.

In our series, all lesions were classified as TASC II C or D lesions, with an average lesion length of 247 mm ± 30 (Figures 1(a), 1(b), 2(a), and 2(b)).

### 3.2. Lesions and Procedural Characteristics

Lesion and procedural characteristics are summarized in Table 3, with 100% of patients having lesions classified as TASC II C or D. Lesion length had a mean of 247 mm ± 30, with 85% (*n* = 11) having severe calcification.

In our series, we had 100% (*n* = 13) successful popliteal artery puncture and access, as well as 100% (*n* = 13) successful SFA recanalization and stenting (Figures 1(c), 1(d), and 2(c)). 46% (*n* = 6) of the cases were done in the prone position after failure of recanalization through a femoral access in the supine position at a previous procedure, while 54% (*n* = 7) were done in the supine position, with concomitant femoral and retrograde popliteal access. Introducer size used for the popliteal access was at a mean of 5.6 F ± 0.7 with a labelled stent diameter at a mean of 6.125 mm ± 0.6.

23% (*n* = 3) patients had deployment of a vascular closure device at the RPA with an extravascular plug placement.

### 3.3. Complications

Complications at the access site are summarized in Table 4, with a total of 15% (*n* = 2) of patients developing a complication at the popliteal access site.

In one case, a hematoma was seen on follow-up ultrasonography, with no sign of an AV shunt or pseudoaneurysm. This hematoma needed no further intervention except for serial US until resolution. In another case, the incorrect deployment of the vascular closure device led to the intravascular deployment of the extravascular plug. This caused an acute thrombosis of the vessel that was discovered postprocedure and promptly treated by a subsequent femoral-popliteal bypass.

## 4. Discussion

Subintimal arterial flossing with antegrade-retrograde intervention (SAFARI) can be useful for completing subintimal recanalization when there is failure to reenter the distal true lumen from an antegrade approach or when there is limited distal target artery available for reentry [11]. In this study, we are reporting our initial experience with SFA recanalization using a popliteal artery approach. RPA was only utilized after failure of recanalization using a femoral access, whether with a contralateral retrograde femoral access or with an ipsilateral antegrade femoral access.

Recommendation for treatment of TransAtlantic Inter-Society Consensus (TASC) C and D lesions has been traditional bypass surgery [12, 13]. However, patients with complex lesions that fit into the TASC C or D classification often present with critical limb ischemia, rest pain, and tissue loss, as well as extensive comorbidities that make them at high risk under traditional open surgical treatment [14]. Improvements in endovascular therapy have made it possible to treat such complex occlusive lesions with less risk [15, 16].

In our series, 13 patients had CTO of the SFA classified as TASC C and D lesions, of which 10 presented with critical limb ischemia. All of those patients failed endovascular treatment by femoral access, and no surgical treatment was done, due to either surgical contraindications or patient preference despite adequate explanation.

Our results were encouraging with excellent technical success and significant improvement of ABI (Table 2); only one severe complication was related to the closure device deployment. A possible explanation for this success rate is that the distal occlusion stump is usually tapered therefore increasing the likelihood of an endoluminal retrograde recanalization, when compared to the proximal, more resistant fibrous cap [17–19]. Despite this fact, in some cases, subintimal passage of the wire could not be avoided; in such cases, the previous attempts of recanalization through a femoral access would allow easy reentry to the true lumen [20]. And when all of this fails, a reentry device has been described to be used successfully and safely to allow for true lumen reentry [21].

Complications at the access site can occur during the arterial access where puncture of the popliteal vein can result in an AV shunt or during hemostasis, at the end of the procedure, where failure of complete hemostasis can result in pseudoaneurysms or hematomas. To decrease the risk of complications related to the arterial puncture, all popliteal arterial access was done under ultrasound guidance. Both sonographic and fluoroscopic methods (roadmap technique after contrast medium injection) have been utilized successfully for popliteal artery puncture, with a recent predominance for puncturing the popliteal artery with the patient in the supine position [22, 23].

In our case series, a vascular closure device (VCD) was used in 3 patients since closure devices were an attractive alternative to manual compression. We chose an entirely extravascular VCD because of the smaller size of the popliteal artery when compared to the common femoral artery and since the ExoSeal VCD has been found to be used successfully and with a low complication rate in puncture sites other than the CFA [2, 24, 25]. In one of the cases, a plug embolization into the popliteal artery led to a complete occlusion of the popliteal artery, requiring a femoropopliteal bypass. Since that case, no VCD has been used; as in many case series prior to ours, only 3 to 10 minutes of manual compression were needed to achieve hemostasis with minimal complications [26]. To avoid the need for a VCD, we needed to decrease the introducer sheath size. One of the techniques used to decrease the need for a large introducer sheath at the popliteal access is to use the popliteal artery access for lesion crossing only. The remainder of the procedure, balloon dilatation and stent placement, is continued from a femoral access. This bidirectional approach would definitely make the procedure more invasive with two arterial punctures but with the advantage of theoretical decrease in complication at the popliteal access site. To take things further, even a sheathless method for the retrograde popliteal access was shown to be successful with a decrease in complication rate when compared with a 4 F or a 6 F sheath [27].

In our experience, there is a big advantage in having the patient in the supine position during the popliteal puncture. Patients in the prone position tend to feel fatigue, more so in obese patients and patients with impaired respiratory function [6]. The bidirectional antegrade-retrograde intervention is also easier for the physician when the patient is in the supine position. And having the patient change position midprocedure can be cumbersome and stressful for the patient. Therefore, the ability to puncture the popliteal artery safely with the patient in the supine position is really important in the facilitation of the procedure.

### 4.1. Limitations

This is a retrospective study with a small number of patients evaluated. Further research is needed with larger prospective studies.

## 5. Conclusion

In conclusion, the development of thinner sheaths and low-profile angioplasty devices made retrograde popliteal access a safe and successful technique, which extends the ability to perform endovascular interventions in complex SFA lesions, where the antegrade approach has failed.

We learned from our study that keeping the patient in the supine position and having a concomitant femoral and popliteal access is better than the prone position, with only a retrograde popliteal access, as it is more comfortable for the patient and easier for the physician, not to mention the ability to decrease the sheath size used at the popliteal access with the supine position as the popliteal artery access would be used for crossing the lesion only, and the remainder of the procedure, balloon dilatation and stent placement, would be continued from a femoral access.

Randomized controlled trials which evaluate long-term patency after this specific type of approach have yet to be designed.

## Figures and Tables

**Table 1:** Characteristics of patients.

Demographics	
Limbs treated	13
Male	8 (61.5)
Average age	77.6 ± 7.5
Presentation	
Claudication	3 (23)
Rest pain	10 (77)
Tissue loss	9 (69)
Comorbidities	
CAD	13 (100)
CHF	4 (31)
Smoking	10 (77)
DM	12 (92)
DL	13 (100)
HTN	13 (100)
CKD	9 (69)
CVA	0 (0)

Continuous data are presented as the means ± standard deviation; categorical data are given as the counts (percentage). CAD: coronary artery disease; CHF: congestive heart failure; DM: diabetes mellitus; DL: dyslipidemia; HTN: hypertension; CKD: chronic kidney disease; CVA: cerebrovascular accident.

**Table 2:** Ankle-brachial index, pre- and postprocedure.

	ABI preprocedure	ABI postprocedure
Patient 1	0.3	0.75
Patient 2	0.45	0.8
Patient 3	0.37	0.7
Patient 4	0.6	0.86
Patient 5	0.54	0.9
Patient 6	0.5	0.85
Patient 7	0.4	0.8
Patient 8	0.48	0.95
Patient 9	0.58	0.8
Patient 10	0.61	0.92
Patient 11	0.43	0.78
Patient 12	0.55	0.82
Patient 13	0.31	0.7

**Table 3:** Lesion and procedure characteristics.

Position and access	
Prone with only popliteal access	6
Supine with femoral and popliteal access	7
TASC II classification	
A/B	0 (0)
C	8 (62)
D	5 (38)
Number of runoff vessels	1.75 ± 0.66
Length of lesions in mm	247 ± 30
Calcification	
Mild	0 (0)
Moderate	2 (15)
Severe	11 (85)
Labelled stent diameter	6.125 ± 0.6
Introducer size	5.6 ± 0.7
Closing device use	3 (23)

Continuous data are presented as the means ± standard deviation; categorical data are given as the counts (percentage).

**Table 4:** Complications.

Pseudoaneurysm	0 (0)
Hematoma	1 (8)
AV shunt	0 (0)
Neuropathy	0 (0)
Acute thrombosis	1 (8)
Total	2

Categorical data are given as the counts (percentage).

## Data Availability

Further data can be requested through email.
